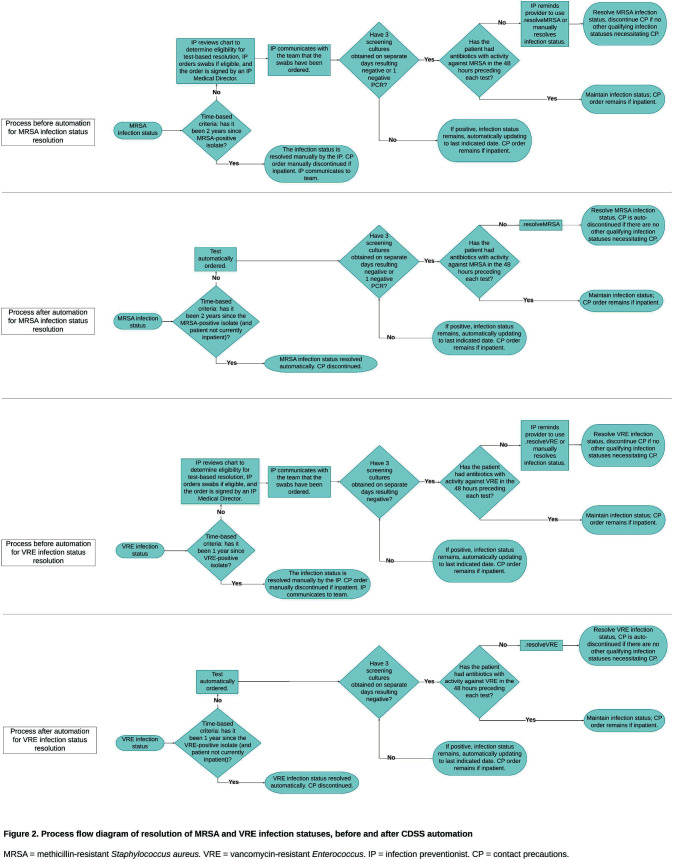# Timesavers: Clinical Decision Support and Automation of MRSA and VRE Deisolation

**DOI:** 10.1017/ash.2024.271

**Published:** 2024-09-16

**Authors:** Molly Paras, Rebecca Craig, Lindsay Germaine, Chloe Green, Zoe Vernick, Jasmine Ha, Erica Shenoy

**Affiliations:** Massachusetts General Hospital; Mass General Brigham; Infection Control; MGB Digital

## Abstract

**Background:** Most healthcare facilities in the US apply contact precautions (CP) for patients with methicillin-resistant Staphylococcus aureus (MRSA) or vancomycin-resistant enterococci (VRE) infection and/or colonization. Most individuals with MRSA or VRE colonization will clear over time; however, frontline clinicians rarely evaluate for discontinuation of CP, resulting in increased burden on infection preventionists (IPs). Automation of time- and test-based evaluation using clinical decision support systems (CDSS) embedded in electronic health records (EHR) may increase evaluation and discontinuation of CP when appropriate, while preserving IP resources. **Methods:** This quality improvement initiative was implemented at Mass General Brigham (MGB), an integrated healthcare system, where patients with MRSA or VRE infection/colonization are identified in the EHR with a corresponding “infection status” and CP applied. Following MGB policy (Figure 1), CDSS features included: 1) automated time-based resolution from 2/15/2023-11/13/2023 and 2) automated ordering of screening assays for patients eligible for test-based evaluation from 6/20/2023-11/14/2023 (Figure 2). Counts of CP discontinuation and automated ordering were performed. IPs at one MGB facility performing manual review of patients self-recorded the time spent evaluating for CP discontinuation. Using these time reports, the average time to complete these tasks and the projected time savings were calculated over the implementation period. **Results:** Four IPs recorded the time to review patients for CP discontinuation, including reviewing recent antimicrobial administration, microbiology results, ordering screening test(s), and contacting the primary team. Twenty-five patient encounters were timed with a mean of 4.7 minutes documented per encounter. Over a 9-month period after initiation of the automated time-based resolution, the monthly mean number of patients with CP for MRSA and VRE which were automatically discontinued was 247 and 100, respectively. Projected IP time savings over the same 9-month period for MRSA and VRE were 174.1 and 70.5 hours, respectively. Over a 5-month period after initiation of automated ordering of MRSA polymerase chain reaction (PCR)/culture, as well as VRE culture for test-based evaluation, the monthly mean number of MRSA culture, MRSA PCR, and VRE culture automatically ordered for patients on CP for MRSA and VRE were 176, 24, and 145, respectively. Projected IP time savings over the same 5-month period for MRSA and VRE were 78.3 and 56.8 hours, respectively.

**Conclusion:** Healthcare systems that enhance their EHR with CDSS to automate CP evaluations may improve frontline clinician workflow, patient flow and bed capacity, while optimizing use of IP resources.

**Figure:**
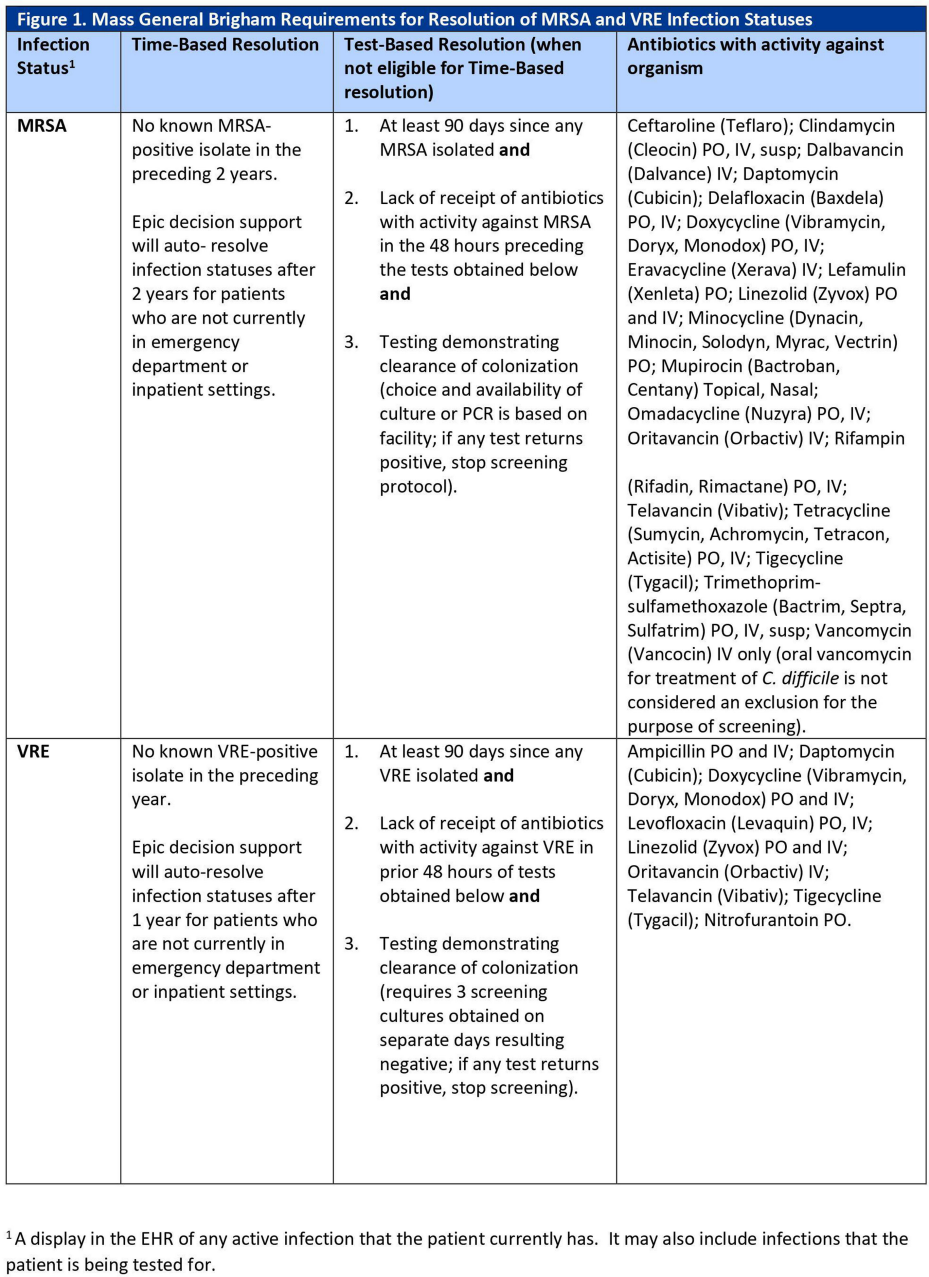

**Figure:**